# Sports Participation and Value of Elite Sports in Predicting Well-Being

**DOI:** 10.3390/sports9120173

**Published:** 2021-12-20

**Authors:** Alfredo Silva, Pedro Sobreiro, Diogo Monteiro

**Affiliations:** 1 Life Quality Research Center (CIEQV), Department of Sport Management, Polytechnic Institute of Santarém, Sport Science School of Rio Maior, 2040-413 Santarém, Portugal; alfredosilva@esdrm.ipsantarem.pt; 2 Research Center in Sport, Health and Human Development (CIDESD), ESECS—Polytechnic of Leiria, 2411-901 Vila Real, Portugal; diogo.monteiro@ipleiria.pt

**Keywords:** elite sport, physical activity, sports participation, sports policy, subjective well-being

## Abstract

This work contributes to an emerging literature focused on the role of physical activity on the subjective well-being of populations. Unlike the existing literature, it proposes an approach that uses algorithms to predict subjective well-being. The aims of this study were to determine the relative importance of sports participation and perceived value of elite sports on the subjective well-being of individuals. A total of 511 participants completed an online questionnaire. The statistical analysis used several machine learning techniques, including three algorithms, Decision Tree Classifier (DTC), Random Forest Classifier (RFC), and Gradient Boosting Classifier (GBC). In the three algorithms tested, sports participation, expressed as the weekly frequency and the time spent engaging in vigorous physical activity, showed a greater importance (between 47% and 53%) in determining subjective well-being. It also highlights the effect of perceived value of elite sport on the prediction of subjective well-being. This study provides evidence for public sport policy makers/authorities and for managers of physical activity and sport development programs. The surprising effect of the perceived value of elite sport on the prediction of subjective well-being.

## 1. Introduction

Increasing sport participation in order to promote citizens’ quality of life, health and well-being has been one of the main goals of the policies implemented by many European governments. The first studies on the subjective well-being of citizens from the perspective of measuring the usefulness of “experience” were developed in 1999 [1,2]. Several factors—such as income, work situation, academic degree, gender, and race—influencing citizens’ subjective well-being have been studied/explored by a number of investigations [1,3,4].

Sports practice has been the subject of attention in recent years; however, studies on the effects of physical activity and sports on the subjective well-being of individuals, compared with the effects of other factors, remains oftentimes unexplored [5] and relatively unusual, e.g., [6,7,8,9,10,11,12,13,14], such that it has been reported that there seems to be a series of gaps, including the possible impact of non-observed variables [3], such as the frequency and weekly duration of physical activity sessions.

The perceived value of elite sports has not received much attention and there is still no clear evidence about the effects of the international sporting success of a country on the subjective well-being of individuals [15].

Based on the aforementioned framework, to determine subjective well-being, this study evaluates the effects of (1) the weekly frequency and duration of sport sessions; and on the other hand, as an innovation in the literature, it uses the (2) perceived value of elite sports as a subjective and individual indicator of the international sporting success of teams and athletes from the country.

This is relevant for two main reasons. The first is related to the promotion of sports participation by the general population: (a) The World Health Organization issued guidelines on physical activity and sedentary behavior [16]; (b) the European Union disseminated Recommended Policy Actions in Support of Health-Enhancing Physical Activity [17], and (c) sports have an increasingly bigger impact on the economies of European countries, with 40% of European citizens (more than 172 million) practicing physical activity and sports at least once a week [18].

The second reason is related to the importance of elite sports in each country, thus justifying the use of the predictor “perceived value of elite sports”: (a) in most modern countries, elite sports have been receiving higher investment and are being considered main vehicles for (i) improving international prestige [19], (ii) raising national unity and pride, and (iii) increasing subjective well-being through the “feelgood factor” after sporting success [15,20,21].

Therefore, what is the relative effect and importance of sports participation and the perceived value of elite sports on the subjective well-being of individuals?

The present study aimed to determine the relative importance of sports participation and perceived value of elite sports on the subjective well-being of individuals through three well-known machine learning techniques: Decision Tree Classifier (DTC), Random Forest Classifier (RFC) and Gradient Boosting Classifier (GBC).

A significant portion of the literature has devoted attention to the identification of determinants of the subjective well-being of individuals [12,22].

### 1.1. Physical Activity and Subjective Well-Being

Different approaches have been adopted over time to investigate the quality of life and well-being of people. In recent years, attention to this topic has grown considerably, with studies analyzing it under different names, such as happiness and satisfaction with life. In general, the topic addresses how people evaluate their lives [23].

Definitions of subjective well-being can be grouped into three domains [24], with this study adopting the one formulated by social research, which defines subjective well-being as “a broad category of phenomena that includes people’s emotional responses, domain satisfactions, and global judgments of life satisfaction” [25] (p. 277), which reflects the current global assessment of the individual about his/her life [24].

There are few studies in the field of sports that have analyzed whether the determinant physical activity and sports has a positive impact on the overall well-being of individuals. It is argued that practicing physical activity and sports is a personal decision that maximizes the rational utility of an individual and must therefore be logically associated with an increase in subjective well-being [10].

The available knowledge has shown a convergence of significant positive effects of sports participation on subjective well-being; it was found that sports participation, both in collective and individual sports, increased subjective well-being [6], with significant effects on men but not significant on women [8] nor on all age groups [11,12]. Recreational and utilitarian walking and cycling were also found to have positive effects on the happiness of individuals [13]. Distinct effects on subjective well-being have been observed when sports comprise higher levels of social interaction (team and partner), higher monthly frequency [7] and exercise intensity and regularity [9].

Surprisingly, the variables weekly frequency and duration of sport sessions have been neglected in previous studies, except [7,9]; however, they are very relevant because they express the cost and effort for individuals and therefore seem to be plausible elements to explain a relationship between sports participation and subjective well-being. Do a higher weekly frequency and longer sessions of physical activity lead to higher levels of subjective well-being? Are there optimal frequency and duration levels that likely maximize levels of subjective well-being?

### 1.2. Perceived Value of Elite Sports and Subjective Well-Being

Several methods can be used to measure international sporting success [26,27]. However, one of the most popular analyses of a nation’s sport performance is the global comparison of countries based on the Olympic medal table because it plays an important role in the media and contributes to individuals forming an idea of the merits of public sport policies and the results of these policies expressed in the perceived value of elite sports in a country.

The perceived value of elite sports recognizes the subjective view of individuals of what constitutes international sporting success in their country and refers to a global and individual summary assessment of the value of a nation’s elite sports in the international context.

**Figure 1:**
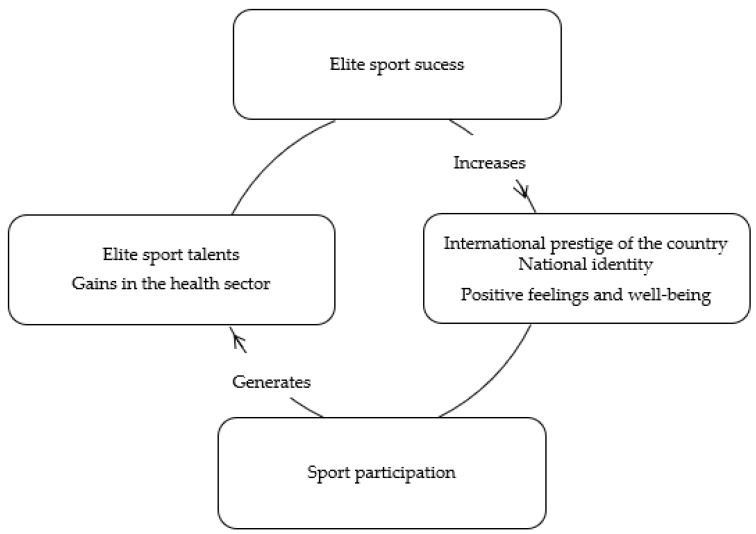
The “virtuous cycle” of sports. Adapted from [27].

The international prestige of the country has guided a philosophy of public investment by the governments of many developed countries (United Kingdom, France, and Australia) in elite sports. The “virtuous cycle” [27] is the concept through which success in elite sports (a) fosters positive feelings and well-being in the population and (b) increases sports participation, leading to advances and gains in the health sector as well as the development of talent in elite sports, once again, leading to the success of elite sports, as shown in Figure 1.

The effects of sporting success on national pride and subjective well-being have not been widely studied [28]; still, the results reflected that sporting success, e.g., in the Olympic Games and in the main international football competitions (FIFA World Cup and UEFA Europa League), marginally affects, in the short term, alleged happiness (subjective well-being) [15]. In agreement with these effects, albeit marginal, it was observed that the athletes’ perceived sporting success led to small positive short-term effects on subjective well-being [29] and that two-thirds of German citizens felt pride and happiness when national athletes achieved success in major international competitions [30].

Thus, government interventions in the sport sector are substantial all over the world. England has the goal of “sustainable improvement in success in international competition, particularly in the sports that matter most to the public, primarily because of the ‘feelgood factor’ associated with winning” [20] (p. 12).

In this context, do the sporting results of elite sports truly have desirable effects on the subjective well-being of citizens, as many politicians believe they have? Is it plausible that the value attributed by the population to elite sports in their country can lead to an increase in the subjective well-being of individuals?

## 2. Materials and Methods

A convenience sample from the population of Portugal was the target population of the study. A total of 567 adequate and usable responses were received—32.6% men and 67.4% women. The mean age was 25.1 ± 9.74 years. Marital status ranged from single, 85.1%, to married, 13.6%, and divorced or widowed, 1.3%. Participants were sent an e-mail with a link to an online questionnaire. The respondents did not receive any monetary compensation for their participation. Respondents were assured that they would remain anonymous and their information confidential before the data collection. The questionnaire respondents were asked about variables such as physical activity [7]; perceived value of elite sports [29]; and subjective well-being [31,32]. Such variables were measured according to what was previously assessed in other studies. Questions regarding sociodemographic details such as age, gender and marital status were also asked. An overview of the variables is provided in Table 1.

In this study, we investigate and compare the performances of several machine learning techniques (DTC, RFC, and GBC) using data collected from a survey representing sports participation and perceived value of elite sport as predictors of subjective well-being. The prediction uses a set of techniques for learning to model the relationship between a set of descriptive characteristics and a target characteristic [33], where the assessment of accuracy is based on the relationship between correct predictions and the total number of predictions [34]. In this study, we considered the algorithms available in scikit-learn [35].

DTC are tree-shaped structures which represent groups of decisions to generate rules [36], where their main advantage is that they are simple to understand and there is no need to normalize the data [37]. RFC uses the same principle of DTC to build a collection of de-correlated trees and averages them [38], reducing the limitation of the DTC to overfit to the training data [39]. GBC uses several weak learners based on decision trees [40] and the models are added in sequence combining the weak learners to create a strong prediction [41].

For evaluation of the models, we used two main methods: the holdout method [34,42] and the k-fold cross-validation method [36].

The accuracy in the prediction was obtained for each algorithm based on the confrontation of the prediction against the answered subjective well-being, positive or negative. These values were calculated using a confusion matrix: true positive (TP—predict positive well-being with positive well-being), true negative (TN—predict negative well-being with negative well-being), false positive (FP—predict positive well-being with negative well-being), and false negative (FN—predict negative well-being with positive well-being). The accuracy was calculated as TP/(TP + FP).

We examined the machine learning models and evaluated their performances using the holdout method (70/30) and 10-fold cross-validation method based on their accuracy.

The accuracy is represented in Table 2. The algorithm with the best performance was GBC for both approaches, with 0.90. DTC had the worst performance, with an accuracy of 0.84 in the holdout method and 0.86 in the k-fold method.

## 3. Results

Sports participation variables were identified as the most important predictors of subjective well-being in two of the three algorithms (GBC, SportPart_1 = 31%; DTC, SportPart_2 = 30%) using train test. The variables (SportPart_1 and SportPart_2) were, in all algorithms, stronger predictors of subjective well-being (DTC = 54%, RFC = 53%, GBC = 53%) than the perceived value of elite sports variables were (DTC = 46%, RFC = 47%, GBC = 47%).

The perceived value of elite sports in the RFC model was identified as the most important predictor of subjective well-being (RFC, SportValuePart_2 = 28%), as shown in Table 3.

Considering the k-fold method, the GBC (the most accurate) and RFC algorithms showed that sport participation variables (GBC, SportPart_2 = 26% and SportPart_1 = 25%; RFC, SportPart_2 = 28% and SportPart_1 = 26%) were the strongest predictors of subjective well-being, as shown in Table 4. For the DTC algorithm (the least accurate), the variable with the greatest predictive value of subjective well-being was the perceived value of elite sports (DTC, SportValue_2 = 36%, SportValue_1 = 17%). The differences in predictive accuracy between the holdout method and in k-fold method were between 4% and 6%. The accuracy was higher for the GBC algorithm in both methods.

## 4. Discussion

### 4.1. Physical Activity and Subjective Well-Being

One of the research questions posed herein was related to whether a higher weekly frequency and longer sessions of physical activity led to higher levels of subjective well-being. Research has shown that certain sports participation variables are important predictors of the subjective well-being of individuals [6,7,8,9,10,11,12,13,14].

In the three algorithms tested, sports participation, expressed as weekly frequency and time spent practicing vigorous physical activity, showed a higher importance (between 47% and 53%) in predicting the subjective well-being of the individuals.

This result clearly confirms what was found with regard to the positive effects of physical activity, in terms of frequency and duration, on subjective well-being and life satisfaction [7,9]. A higher weekly frequency and longer session duration led to higher levels of subjective well-being in individuals. This result can be explained from two perspectives. The first is supported by the so-called “virtuous cycle” [27], which argues that there are gains in health, which may be associated with subjective well-being. The second perspective holds that engaging in physical activity involves rational utility for the individual, and therefore, the individual rates physical activity as positive and favorable [10]. The WHO key message “Any amount of physical activity is better than none, and more is better” [16] (p. 7), seems congruent with our results, in such a way that the weekly frequency and duration of sport sessions showed positive effects on the subjective well-being, but the extensive meta-analytic review [43] showed that more exercise was not always better. Differences were found in terms of exercise type, exercise duration and frequency, which may be more effective. Therefore, specialized technicians to conduct physical activity programs are essential to ensure the best results for individuals.

### 4.2. Perceived Value of Elite Sports and Subjective Well-Being

The aim was to determine to what extent the perceived value of elite sports influences the subjective well-being of citizens, as research has shown that the success of athletes and sports teams in international competitions, in addition to the effects on feelings of national pride and international prestige [44], is a general factor of positive feelings in a population [27] and of the subjective well-being of individuals [39]. This feelgood factor after sporting success has been highlighted, even justifying the investments in elite sports by various governments [20].

The present study’s results provide significant support to this idea. The perceived value of elite sports in a country has relevant importance (46% to 53%) in predicting the subjective well-being of individuals.

In previous studies, pride for international sporting success did not have direct results: athletes’ perceived sporting success led to small short-term positive effects on subjective well-being [29] and two-thirds of German citizens felt pride and happiness when national athletes achieved success in major international competitions [30]. Despite this divergence, findings from the present investigation seem to legitimize that the perceived value of elite sports in a country positively and significantly affects individuals’ sense of well-being.

However, there are conflicting results. Pride in international sporting success did not have direct results in terms of gains in subjective well-being [8]. Despite this divergence, the results seem to legitimize the implementation of heavy investment policies in elite sports by governments. The rationale lies in the effects of the so-called “virtuous cycle” [27], which in addition to stimulating sports participation in the population also increases the perceived value of elite sports in the country, which in turn leads to positive feelings of well-being in individuals.

Two theoretical implications can be drawn from the results obtained. The first refers to the confirmation that sports participation with greater regularity and longer sessions positively impacts subjective well-being. This contribution strengthens the knowledge about the role of sports participation. For example, in a large US sample, physical exercise was significantly and meaningfully associated with self-reported mental health burden in the past month. However, more exercise was not always better. Differences were found in terms of exercise type, exercise duration and frequency, which may be more effective clinical targets to reduce the burden of mental health [43].

The second implication results from the theoretical connection found between the perceived value of elite sports and subjective well-being, which will allow the expansion of the research on international sporting success, the perceived value of elite sports and the subjective well-being of individuals. Third, the methodology involving the algorithms used was considered adequate, namely, the GBC algorithm, as it showed results similar to those obtained by several studies published using other methods.

In terms of management, two targets are found for whom results may be of interest: the public sport policy makers/authorities and sport development program managers.

First, the results found provide a boost to growing public policies that seek to promote quality of life and the generalization of participation in physical activity. The evidence that sports participation increases subjective well-being provides support for the prioritization of policies through governments to concentrate efforts on the goal of increasing sports participation at all levels of the population. This posits that the benefits of sport participation should be emphasized in public information campaigns as policy instruments to increase subjective well-being levels. The goal should be to increase the subjective well-being of individuals, stimulating governments to design public policies and programs for sports participation targeting two adult populations: (1) sedentary individuals and (2) individuals who participate in light physical activity. For sedentary individuals, the goal is for them to start participating in sport activities and thereby initiating a process of gains in levels of subjective well-being. For those who practice light physical activity, the goal is to increase the frequency and duration of the sessions, which, as proved, tends to increase levels of subjective well-being.

There is a need to reinforce certain policies targeting high-performance sports. This position had already been advocated [28], encouraging politicians to be aware that all segments of the population benefit equally, in terms of well-being, from the sporting successes obtained in international competitions. Governments should develop sports policies for elite sports, devoting investments in support of national teams and elite athletes with strong chances of achieving international sporting success, which in turn increases the perceived value of elite sports, leading to higher levels of the subjective well-being of citizens. On the other hand, governments can influence the perceived value of sports, developing communication campaigns that show genuine sports achievements, which in turn can lead to an increase in subjective well-being.

The potential limitations of our study likely have an influence on the results and may be related to the sample size, the higher proportion of women and the average age being 25 years. Another limitation could be related to sample size; however, considering that annotated data are a scarce resource and expensive to obtain, a sample size between 100 to 200 is considered acceptable (Figueroa et al., 2012). In the subjective well-being variable, factors such as health/disease status, age, sex, body mass index, income, race, marital status and education were not considered.

In the field of valuing sporting success, future studies may focus on comparisons with other countries and sociodemographic segments, as the national importance of each sport (e.g., soccer in Portugal and soccer in the United States) may differ from country to country. In addition, future studies should seek to further explore the link between gender, age, sport participation and perceived value of elite sports, using a more balanced sample and testing subsamples of different genders and similar levels of sport activity.

## 5. Conclusions

This study enriches the literature focused on factors that contribute to the prediction of factors that contribute to the prediction of citizens’ subjective well-being. The results also allowed us to reach three key conclusions. Firstly, the sport participation of citizens, in terms of regularity and duration of sessions, has a positive and direct effect, creating a relevant predictor of subjective well-being. This conclusion is in line with the World Health Organization’s [16] recommendations on guidelines on physical activity and sedentary behavior, specifically with the first two key messages: (1) physical activity is good for hearts, bodies and minds; (2) any amount of physical activity is better than none, and more is better. Secondly, this study provided compelling evidence that, in addition to sport participation, the perceived value of a country’s performance in elite sports alone is an important predictor of the population’s subjective well-being. Third, it supports and legitimizes the “virtuous cycle of sports” idea proposed by Grix and Carmichael, which argues that elite sport success leads to positive feelings and well-being for citizens.

## Figures and Tables

**Table 1:** Variable definition, descriptive statistics, and measurement.

Variable	Description	Scale	Mean (SD *)	Source
Physical activity (SportPart_1; SportPart_2)	(1) How often do you exercise or play sport? (in last month) (1 = Never; 2 = 1 to 3 times a month; 3 = 1 to 2 times a week; 4 = 3 to 4 times a week; 5 = 5 times a week or more). (2) In general, on days when you do vigorous physical activity, how much time in total do you usually spend at it? (1 = Never do vigorous physical activities; 2 = 30 min or less; 3 = 31 to 60 min; 4 = 61 to 90 min; 5 = 91 to 120 min; 6 = More than 120 min).	Metric	(1) 2.83 (1.27) (2) 3.1 (1.29)	Adapted from [7]
Perceived value of elite sport (SportValue_1; SportValue_2)	(1) Currently, in the international context, the position of Portuguese high-competition sport is: (1 = Extremely bad, 2 = Very poor, 3 = Bad, 4 = Fair, 5 = Good, 6 = Very good, 7 = Extremely good); (2) And relatively 10 years ago, in the international context, globally, the position of Portuguese high competition sport is: (1 = Extremely worse than 10 years ago; 2 = Much worse; 3 = Worse; 4 = Equal; 5 = Best; 6 = Much better; 7 = Extremely better than 10 years ago)	Ordinal	(1) 5.4 (1.11) (2) 4.6 (1.2)	Adapted from [29]
Subjective well-being (SWB)	Satisfaction with Life Scale: Overall, I am satisfied with my life. (0 = Not at all satisfied to 10 = Extremely satisfied) Negative = 0; Positive =1	Ordinal	8.16 (1.91)	Adapted from [31,32]
Gender (Gender)	(1 = Female; 2 = Male)	Dummy	1.32 (0.46)	
Age (Age)	Age in years	Metric	25.1 (9.74)	
Maritage status (MaritalStatus)	(1 = Single, not married; 2 = Couple; 3 = Divorced; 4 = In union of fact; 5 = Widow)	Reference category	1.29 (0.79)	

* SD—standard deviation.

**Table 2:** Accuracy train/test and k-fold for each algorithm.

Algorithm	DTC	RFC	GBC
Holdout	0.84	0.88	0.90
K-fold	0.86	0.88	0.90

DTC—Decision Tree Classifier; RFC—Random Forest Classifier; GBC—Gradient Boosting Classifier.

**Table 3:** Importance of the variables for each algorithm predicting the well-being using train test.

DTC		RFC		GBC	
Variable	Importance (%)	Variable	Importance (%)	Variable	Importance (%)
SportPart_2	30	SportValue_2	28	SportPart_1	31
SportValue_2	26	SportPart_2	27	SportValue_1	29
SportPart_1	24	SportPart_1	26	SportPart_2	22
SportValue_1	20	SportValue_1	19	SportValue_2	18

Note: Sport Participation = SportPart_1 and SportPart_2; Perceived Value of Elite Sport = SportValue_1 and SportValue_2. DTC—Decision Tree Classifier; RFC—Random Forest Classifier; GBC—Gradient Boosting Classifier.

**Table 4:** Importance of the variables for each algorithm predicting the well-being using k-fold.

DTC		RFC		GBC	
Variable	Importance (%)	Variable	Importance (%)	Variable	Importance (%)
SportValue_2	36	SportValue_2	29	SportPart_2	26
SportPart_2	26	SportPart_2	28	SportPart_1	25
SportPart_1	21	SportPart_1	23	SportValue_2	24
SportValue_1	17	SportValue_1	20	SportValue_1	24

Note: Sport Participation = SportPart_1 and SportPart_2; Perceived Value of Elite Sport = SportValue_1 and SportValue_2. DTC—Decision Tree Classifier; RFC—Random Forest Classifier; GBC—Gradient Boosting Classifier.

## Data Availability

Not applicable.
